# Reversible Subacute Liver Failure Following Revisional Malabsorptive Bariatric Surgery: A Case Report

**DOI:** 10.7759/cureus.112698

**Published:** 2026-07-15

**Authors:** Sofia Prada, Rita Penaforte, Beatriz Sá Pereira, Marinela Major, Fernando Aldomiro

**Affiliations:** 1 Faculty of Medicine, Universidade de Lisboa, Lisboa, PRT; 2 Internal Medicine, Unidade Local de Saúde (ULS) Amadora/Sintra, Lisboa, PRT

**Keywords:** bariatric surgery, malabsorption, metabolic dysfunction-associated steatotic liver disease (masld), single-anastomosis duodeno-ileal bypass with sleeve gastrectomy (sadi-s), subacute liver failure, surgical reversal

## Abstract

Bariatric surgery is an effective treatment for obesity and metabolic dysfunction-associated steatotic liver disease (MASLD). Although most patients experience improvement in hepatic steatosis and fibrosis after surgery, severe hepatic decompensation has rarely been reported following malabsorptive procedures. Early recognition of potentially reversible causes of liver failure is essential to prevent progression to irreversible hepatic injury or liver transplantation.

We report the case of a 54-year-old woman with obesity-related MASLD who underwent revisional single-anastomosis duodeno-ileal bypass with sleeve gastrectomy (SADI-S) following weight regain after Roux-en-Y gastric bypass. Ten months after surgery, she developed progressive hypoalbuminemia, recurrent hyperammonemia, coagulopathy and worsening porto-systemic encephalopathy culminating in grade IV encephalopathy requiring intensive care admission and invasive mechanical ventilation. Extensive investigation excluded alternative causes of acute hepatic deterioration. Despite six weeks of intensive conservative management, including optimized nutritional rehabilitation with high-protein enteral supplementation, vitamin and micronutrient replacement, correction of electrolyte disturbances, and standard medical therapy for hepatic encephalopathy (lactulose and rifaximin), hepatic synthetic function continued to deteriorate. Following multidisciplinary discussion, surgical reversal of the malabsorptive anatomy was undertaken. Liver biopsy demonstrated advanced chronic liver disease with cholestatic changes, macrovesicular steatosis and severe secondary hepatic siderosis. Neurological improvement was observed within the first postoperative days, with complete resolution of hepatic encephalopathy and normalization of serum ammonia levels by postoperative day five, followed by progressive recovery of hepatic synthetic function.

This case highlights persistent malabsorption as a reversible driver of subacute liver failure in patients with limited hepatic reserve following revisional bariatric surgery. Recognition of this rare complication is essential because timely surgical reversal may interrupt ongoing metabolic injury, restore hepatic function and prevent progression to irreversible liver failure or liver transplantation.

## Introduction

Bariatric surgery is an established treatment for severe obesity and its associated metabolic complications. In patients with metabolic dysfunction-associated steatotic liver disease (MASLD), sustained weight loss following bariatric surgery has consistently been associated with resolution of steatohepatitis, regression of fibrosis, and reduced long-term liver-related morbidity [1-3].

Despite these well-established benefits, severe hepatic dysfunction has rarely been reported after bariatric procedures with a predominant malabsorptive component. Although uncommon, progressive liver failure following these procedures represents a potentially life-threatening complication that may ultimately require liver transplantation if not recognised promptly [4,5].

The mechanisms underlying hepatic decompensation remain incompletely understood and are likely multifactorial. Current evidence, derived primarily from case reports, small case series, and pathophysiological studies, suggests that persistent protein-calorie malnutrition, micronutrient deficiencies, altered enterohepatic physiology, and sustained catabolic stress may impair hepatic protein synthesis and regeneration. Simultaneously, increased free fatty acid flux, mitochondrial dysfunction, oxidative stress, and chronic inflammatory activation may further compromise hepatocellular function, particularly in patients with pre-existing MASLD or advanced fibrosis [6-8]. Although the relative contribution of each of these mechanisms remains uncertain, they are likely to act synergistically, ultimately exceeding hepatic functional reserve and promoting progressive hepatic decompensation.

Because this entity is exceptionally rare, recognition is frequently delayed and management remains poorly defined. Early identification of potentially reversible causes of hepatic dysfunction is therefore essential to prevent progression to advanced liver failure [9].

We report the case of a woman with advanced MASLD who developed subacute liver failure 10 months after revisional malabsorptive bariatric surgery. Failure of intensive nutritional and medical therapy prompted surgical reversal of the malabsorptive anatomy, resulting in progressive neurological and biochemical recovery. This case supports persistent malabsorption as a reversible driver of hepatic decompensation and highlights surgical reversal as a potential liver-directed therapeutic strategy in carefully selected patients.

## Case presentation

A 54-year-old woman with a history of morbid obesity (maximum body weight 142 kg; body mass index [BMI] 55 kg/m²) underwent Roux-en-Y gastric bypass in 2010, achieving substantial initial weight loss. Her medical history was significant for dyslipidemia and MASLD, with no previous episodes of hepatic decompensation.

Over subsequent years, progressive weight regain prompted revisional bariatric surgery. In October 2023, she underwent conversion to single-anastomosis duodeno-ileal bypass with sleeve gastrectomy (SADI-S), reportedly configured with a 300-cm common channel. The immediate postoperative course was uneventful, and she remained under regular bariatric follow-up while receiving routine postoperative nutritional supplementation according to institutional protocol, including multivitamin, trace element, and vitamin replacement.

Approximately 10 months after surgery, she developed progressive asthenia, anorexia, nausea, peripheral oedema, and intermittent episodes of confusion. Although complete longitudinal postoperative weight measurements were unavailable, outpatient laboratory investigations demonstrated progressive hypoalbuminaemia, worsening coagulopathy, and recurrent episodes of severe hyperammonaemia, with a peak ammonia concentration of 355 µmol/L. Together with the subsequent clinical course, these findings were consistent with progressive hepatic synthetic dysfunction in the setting of severe postoperative malabsorption.

In September 2024, she was admitted to hospital because of progressive neurological deterioration. During admission, her level of consciousness declined rapidly, progressing to grade IV porto-systemic encephalopathy (Glasgow Coma Scale 7-9), requiring endotracheal intubation, invasive mechanical ventilation, and admission to the intensive care unit.

Laboratory investigations confirmed severe hepatic synthetic dysfunction, including profound hypoalbuminaemia (nadir 1.2 g/dL), persistent coagulopathy (international normalised ratio consistently ≥1.5), recurrent hypoglycaemia, electrolyte disturbances, and severe hyperammonaemia. Liver disease severity corresponded to Child-Pugh class C (12 points) and a model for end-stage liver disease with sodium (MELD-Na) score of 22 [10,11].

**Figure 1:**
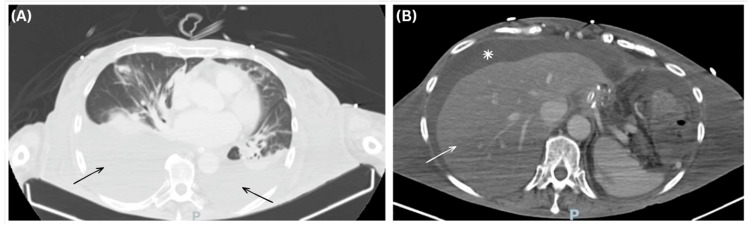
Contrast-enhanced CT demonstrating complications associated with severe hepatic decompensation. (A) Large bilateral pleural effusions (black arrows) with adjacent compressive atelectasis. (B) Hepatomegaly with diffuse hepatic steatosis (white arrow) and surrounding ascites (*).

Transient elastography demonstrated advanced fibrosis (liver stiffness 15.7 kPa) with severe steatosis (controlled attenuation parameter 310 dB/m). Abdominal computed tomography showed hepatomegaly with diffuse steatotic infiltration, moderate ascites, and bilateral pleural effusions (Figure 1). Diagnostic paracentesis confirmed uncomplicated transudative ascites without evidence of spontaneous bacterial peritonitis.

An extensive diagnostic evaluation excluded alternative causes of hepatic deterioration. Reduced ceruloplasmin and alpha-1 antitrypsin concentrations were considered secondary to impaired hepatic synthetic function, as clinical, biochemical, and histological findings were not compatible with Wilson disease or alpha-1 antitrypsin deficiency. Viral hepatitis (hepatitis B virus and hepatitis C virus), autoimmune liver disease (antinuclear, anti-smooth muscle, antimitochondrial and anti-liver-kidney microsomal antibodies), and other metabolic liver diseases were excluded.

Despite six weeks of intensive conservative management, including individualized enteral nutritional rehabilitation, vitamin and micronutrient supplementation, correction of electrolyte abnormalities, and standard medical therapy for hepatic encephalopathy with lactulose and rifaximin, hepatic synthetic function failed to show sustained improvement and neurological recovery remained incomplete. Given the absence of an alternative reversible cause of liver failure and the progressive deterioration despite prolonged optimized medical and nutritional therapy, a multidisciplinary team comprising hepatobiliary surgeons, hepatologists, intensivists, and nutrition specialists recommended surgical reversal of the malabsorptive anatomy.

In October 2024, the patient underwent surgical reconstruction consisting of a duodeno-jejunal bypass with ileal interposition configured as a Roux-en-Y, thereby restoring proximal intestinal absorptive capacity, together with cholecystectomy. Although the original operative report described a 300-cm common channel, intraoperative measurement during the revisional procedure demonstrated an effective common channel of approximately 210 cm, confirming marked functional malabsorption. The reason for this discrepancy could not be definitively established from the available operative records. A wedge liver biopsy was obtained during the procedure.

Histopathological examination demonstrated advanced chronic liver disease characterised by portal and porto-portal fibrotic expansion, ductular reaction, cholestatic features, and macrovesicular steatosis involving approximately 10% of hepatocytes. Marked secondary hepatic siderosis (Scheuer grade 4/4) was also present. Iron studies and HFE genotyping were not available; however, the absence of histological features suggestive of hereditary haemochromatosis, together with the clinical context of severe hepatic synthetic dysfunction and malnutrition, supported secondary hepatic siderosis. No histological evidence of Wilson disease, alpha-1 antitrypsin deficiency, or other primary metabolic liver disorders was identified.

Following surgical reversal, the patient experienced rapid neurological recovery with complete resolution of hepatic encephalopathy, normalisation of serum ammonia concentrations, and progressive improvement of hepatic synthetic function. Albumin levels increased steadily, coagulation parameters normalised, and both Child-Pugh and MELD-Na scores improved substantially during follow-up. She was discharged in stable clinical condition under multidisciplinary follow-up with nutritional rehabilitation and hepatology surveillance.

The chronological evolution of the principal laboratory parameters is summarised in Table 1.

**Table 1:** Chronological evolution of laboratory parameters in a patient with subacute liver failure following revisional SADI-S. Only laboratory values explicitly documented in the medical record are reported. The table demonstrates severe hyperammonemia and hepatic synthetic failure during the malabsorptive phase, followed by progressive biochemical recovery after surgical reversal and restoration of intestinal absorptive capacity. Abbreviations: ICU, intensive care unit; SADI-S, single-anastomosis duodeno-ileal bypass with sleeve gastrectomy; INR, international normalised ratio; MELD, model for end-stage liver disease

Parameter (units)	Reference range	Admission Day 0	ICU admission Day 2	Immediately before reversal surgery Day 42; Week 6	Discharge Day 77
Albumin (g/dL)	3.97-4.94	1.49	1.88	3.49	2.75
INR	< 1.2	2.3	1.9	1.3	1.2
Total bilirubin (mg/dL)	< 1.2	2.82	2.70	0.65	0.41
Direct bilirubin (mg/dL)	< 0.2	1.95	1.90	0.37	0.22
Ammonia (mmol/L)	11-51	355	187	15	15
Sodium (mmol/L)	135-145	135	137	139	141
Creatinine (mg/dL)	0.5-0.9	1.13	0.32	0.30	0.36
Encephalopathy grade		2	4	2	0
Child-Pugh [10]		C (11 points)	C (12 points)	B (8 points)	A (6 points)
MELD-Na [11]		22	19	10	8

## Discussion

Bariatric surgery is widely recognised as one of the most effective therapeutic interventions for obesity and MASLD. Most patients experience sustained improvement in hepatic steatosis, resolution of steatohepatitis, regression of fibrosis, and a reduction in liver-related complications following substantial weight loss [1-3]. The occurrence of progressive liver failure after bariatric surgery is therefore paradoxical and remains an exceptionally uncommon complication.

Published reports suggest that severe hepatic dysfunction occurs predominantly after procedures with a marked malabsorptive component [4,5]. Although the precise incidence is unknown, the available evidence suggests that persistent malabsorption and its metabolic consequences, rather than the specific bariatric procedure itself, are the principal drivers of hepatic injury. The underlying pathophysiological mechanisms are likely multifactorial. Persistent protein-calorie malnutrition and micronutrient deficiencies impair hepatocellular metabolism and regeneration, while rapid weight loss and sustained catabolism increase peripheral lipolysis and hepatic free fatty acid delivery, promoting mitochondrial dysfunction, oxidative stress, impaired lipid export, and progressive hepatocellular injury [6-8]. In patients with pre-existing MASLD, these metabolic insults may exceed hepatic functional reserve, resulting in progressive hepatic synthetic dysfunction. Whether revisional bariatric procedures confer a greater risk than primary malabsorptive operations remains uncertain. Revision surgery may theoretically increase susceptibility to severe malnutrition because of greater anatomical complexity, previously altered gastrointestinal anatomy, and cumulative malabsorptive effects. However, current evidence is insufficient to establish revisional surgery as an independent risk factor for hepatic failure, as the literature is largely limited to isolated case reports and small series. In the present case, conversion of a previous Roux-en-Y gastric bypass to SADI-S may have contributed to a particularly vulnerable nutritional and metabolic state.

Our patient's clinical course is consistent with this proposed mechanism. Progressive hypoalbuminaemia, recurrent hyperammonaemia, coagulopathy, and porto-systemic encephalopathy developed over several weeks in the absence of an alternative precipitating hepatic insult. Furthermore, the lack of sustained improvement despite six weeks of intensive nutritional rehabilitation and standard medical therapy for hepatic encephalopathy suggests that persistent malabsorption was a major contributor to the ongoing hepatic decompensation.

An additional finding was severe secondary hepatic siderosis. Iron accumulation has been associated with oxidative hepatocellular injury and accelerated progression of chronic liver disease, although its precise pathogenic role in postoperative malnutrition remains uncertain [12]. In our patient, siderosis was more likely an aggravating factor than the primary cause of hepatic failure.

The most compelling observation was the rapid clinical and biochemical recovery following restoration of intestinal absorptive capacity. Complete resolution of hepatic encephalopathy and normalization of serum ammonia concentrations were achieved by postoperative day five, followed by progressive recovery of hepatic synthetic function. This improvement was sustained throughout 12 months of follow-up, with no recurrence of hepatic encephalopathy or further episodes of hepatic decompensation. Although previous reports have described patients requiring liver transplantation or experiencing fatal outcomes despite intensive medical treatment [4,5], our case suggests that timely correction of the underlying anatomical abnormality may restore hepatic function and provide durable clinical benefit in carefully selected patients before irreversible liver failure develops.

This case also highlights the importance of early multidisciplinary management. In patients presenting with progressive hepatic dysfunction after malabsorptive bariatric surgery, clinicians should maintain a high index of suspicion for malnutrition-related liver injury once other causes of hepatic decompensation have been excluded. Early collaboration between hepatologists, bariatric surgeons, intensivists, and nutrition specialists is essential to identify patients who may benefit from surgical reversal before progression to advanced liver failure [9].

Although a single case cannot establish causality, it provides clinically relevant evidence supporting persistent malabsorption as a potentially reversible contributor to hepatic decompensation. Several limitations should be acknowledged. Detailed longitudinal nutritional data, including serial nutritional biomarkers, postoperative weight trajectory, and complete information regarding nutritional supplementation, were unavailable. Furthermore, the relative contribution of persistent malabsorption, altered gastrointestinal anatomy, and pre-existing advanced MASLD to the development of hepatic decompensation cannot be determined with certainty. Nevertheless, the marked and sustained clinical recovery following surgical reversal, maintained throughout 12 months of follow-up, supports consideration of persistent malabsorption as an important therapeutic target in carefully selected patients. Greater awareness of this rare complication may facilitate earlier diagnosis, prompt nutritional intervention, and timely surgical reversal, potentially preventing irreversible liver failure and the need for liver transplantation.

## Conclusions

Although bariatric surgery is generally associated with improvement of MASLD, severe hepatic decompensation may rarely occur following procedures with a marked malabsorptive component. Recognition of persistent malabsorption as a potentially reversible driver of liver failure is essential, particularly in patients with limited hepatic reserve.

Our case demonstrates that restoration of intestinal absorptive capacity can be associated with marked neurological and biochemical recovery after failure of conservative management. Early multidisciplinary evaluation is therefore essential to identify patients who may benefit from individualized management. In carefully selected patients with progressive hepatic decompensation despite optimized nutritional and medical therapy, surgical reversal should be considered on a case-by-case basis before progression to irreversible liver failure or the need for liver transplantation.
